# Enhancing Healthcare Accountability for Administrators: Fostering Transparency for Patient Safety and Quality Enhancement

**DOI:** 10.7759/cureus.66007

**Published:** 2024-08-02

**Authors:** Tatsuya Fukami

**Affiliations:** 1 Patient Safety Division, Shimane University Hospital, Izumo, JPN

**Keywords:** shared decision making, psychological safety, quality improvement in healthcare, patient safety, organizational transparency

## Abstract

Transparency in healthcare organizations is essential for creating a culture of patient-centered care where patients are respected, informed, and actively engaged in their health and well-being. Organizational transparency is a crucial element in healthcare, enhancing patient safety and quality improvement. Transparency involves open communication about healthcare organizations’ performance, outcomes, and processes, leading to improved accountability, trust, and patient engagement. Transparent organizations prioritize patient-centered care, involving patients in decision-making and fostering shared mental models between healthcare providers and patients. Psychological safety is vital for organizational transparency. Patient safety reporting systems play a key role in transparency, allowing anonymous reporting of safety concerns and incidents. These systems facilitate early risk identification, continuous improvement, and compliance with regulatory requirements. Transparency in reporting encourages a culture of openness, learning from near misses, and addressing systemic issues and human errors. It aligns with ethical principles, potentially mitigating legal challenges. This review synthesizes key themes, including the importance of patient-centered care, the role of psychological safety in fostering transparency, and the effectiveness of patient safety reporting systems.

## Introduction and background

Organizational transparency in healthcare is a critical component of efforts to enhance patient safety and quality improvement [1]. Recent advancements in healthcare transparency have significantly impacted patient safety and quality improvement. Various studies have highlighted the positive effects of transparency on healthcare outcomes. For instance, Wolf and Hughes [2] demonstrated that hospitals with higher levels of transparency had significantly lower rates of medical errors and adverse events. Donelan et al. [3] found that transparency in healthcare organizations led to higher patient satisfaction and trust in healthcare providers. Transparency in healthcare organizations is essential for creating a culture of patient-centered care where patients are respected, informed, and actively engaged in their health and well-being [4,5]. These studies underscore the growing recognition of transparency as a vital component of effective healthcare delivery.

Despite these advancements, there remain substantial gaps in our understanding of how transparency can be systematically implemented across different healthcare settings. Previous research has predominantly focused on specific aspects of transparency, such as error reporting or patient access to medical records. However, there is a need for a comprehensive review that synthesizes the various dimensions of transparency and their collective impact on patient care. The objective of the present study is to fill this research gap by conducting a thorough review of the literature on transparency in healthcare. This review aims to understand how transparency contributes to patient safety and quality improvement. By synthesizing existing evidence, the study seeks to inform future research, policy development, and practice guidelines aimed at enhancing transparency in healthcare organizations. Addressing this gap is significant for several reasons. First, it will provide a more holistic understanding of transparency and its multifaceted impacts on healthcare delivery. Second, it will identify best practices and strategies for implementing transparency in various healthcare contexts. Lastly, this study will contribute to the development of a patient-centered care culture where patients are informed, respected, and actively engaged in their health and well-being. Through these contributions, the study aims to enhance the overall quality and safety of healthcare.

## Review

Improved accountability

Transparent organizations are more accountable to patients, staff, and the public. When information about performance and outcomes is readily available, healthcare providers and administrators are more likely to be held accountable for their actions and decisions. Certainly, transparency in healthcare organizations plays a crucial role in fostering improved accountability. Transparent organizations encourage open communication [6]. When information about performance, outcomes, and decision-making processes is easily accessible, it facilitates clear and honest communication between healthcare providers, staff, patients, and the public. Access to transparent data allows stakeholders to make informed decisions [7]. Patients can make choices based on the performance and outcomes of healthcare providers, and administrators can use data to make evidence-based decisions for the benefit of the organization. Transparent reporting of performance metrics allows for continuous monitoring. Performance metrics in healthcare can vary widely depending on the aspect of care being measured.

Clinical outcomes include mortality rates (e.g., hospital mortality rate and surgical mortality rate), complication rates (e.g., surgical complications and infections), and disease-specific outcomes (e.g., cancer remission rates and diabetes control metrics). Patient safety focuses on adverse events such as falls and medication errors. Patient satisfaction is measured through surveys and feedback, assessing patient experience, communication with healthcare providers, and overall satisfaction with care received. Operational efficiency, financial performance, and quality of care enable healthcare providers and administrators to track their performance against established benchmarks, identify areas for improvement, and take corrective actions. When patients and the public can easily access information about outcomes, it incentivizes healthcare organizations to maintain high standards and work toward continuous improvement [8]. By openly sharing information, healthcare providers demonstrate a commitment to adhering to regulations and standards, reducing the risk of legal and regulatory issues. Internal operations also benefit from transparency [9]. Staff members within a healthcare organization are more likely to be held accountable for their actions when there is transparency regarding their roles, responsibilities, and performance expectations. Transparency in healthcare organizations creates an environment where information is accessible, decisions are well informed, and stakeholders are held accountable for their actions. This leads to better overall performance, improved quality of care, and increased trust among patients and the public. Accountability and transparency are closely linked concepts, especially in governance and organizational contexts. Transparency can be measured through indicators such as the ease with which stakeholders can access relevant information, the extent of organizational disclosures about activities, finances, and decision-making processes, and the effectiveness of soliciting and responding to stakeholder feedback.

Enhanced trust and patient engagement

Transparency encourages open communication between patients and healthcare providers. When patients have access to information, they may feel more comfortable discussing their concerns, asking questions, and providing feedback, leading to a more collaborative and communicative healthcare relationship. Transparent organizations promote shared decision-making, where patients and healthcare providers collaborate to determine the most appropriate course of action [10]. Establishing psychological safety between medical staff and patients is fundamental to fostering successful shared decision-making. Shared decision-making refers to a collaborative process wherein medical providers and patients collaborate to determine the best course of action for care. This approach encourages mutual understanding and cooperation, ensuring that decisions align with the patient’s preferences and values [11-13].

For instance, implementing decision aids to promote shared decision-making can be challenging if there is not a solid shared mental model between clinicians and patients. Shared mental models enable healthcare professionals to perceive patients not only as recipients of care but also as partners, decision-makers, collaborators, and integral members of inter-professional healthcare teams. This mindset facilitates patient-centered care by enabling healthcare professionals to empathize with and honor the patient’s viewpoint and choices. Shared mental models are crucial in fostering collaboration among healthcare providers and contributing to shared decision-making.

Informed consent serves as a fundamental pillar of patient-centered care, respecting patients’ autonomy and rights by ensuring their active participation in healthcare decisions. It involves patients fully comprehending the risks, benefits, and alternatives associated with their treatment options. Healthcare providers have the responsibility of providing essential information transparently to facilitate informed consent. It is crucial to differentiate between informed consent, informed choice, and shared decision-making. Informed consent involves physicians explaining and recommending the best care option(s) based on medical examinations and clinical evidence, which the patient comprehends and accepts. Informed choice and shared decision-making similarly involve medical examinations and clinical evidence, with physicians explaining and recommending the benefits and risks of care options. However, shared decision-making further involves patient-physician collaboration, considering the patient’s hopes, wishes, thoughts, beliefs, and social background to ensure treatment plans align with their values and preferences.

Conflict, when handled constructively, can lead to improved outcomes, fresh perspectives, and business growth. Healthy conflict is characterized by mutual respect and trust, where participants can express differing opinions without fear of bullying or denigration. Encouraging open communication, setting clear expectations, embracing diversity, fostering healthy debate, and leading by example is vital for fostering healthy conflict within teams, leading to increased productivity, engagement, innovation, creativity, and collaboration [14].

Patients often experience fear and anxiety when seeking medical care and require psychological safety. Patient engagement involves involving patients in their care, providing them with information and resources to make informed decisions, and allowing them to participate in care policy [15]. This can be achieved through patient education and empowerment, effective patient-provider communication, and encouraging patient self-management. Patient engagement is integral to patient-centered care, aiming to understand and meet each patient’s individual needs and preferences, ultimately improving health outcomes and patient satisfaction [16,17]. Patient and family engagement has been integrated into the Global Patient Safety Action Plan 2021-2030 as a key strategy for reducing avoidable harm in healthcare [18]. Psychological safety plays a crucial role in patient engagement, as patients are more likely to feel comfortable and express their concerns openly when they perceive psychological safety in their interactions with healthcare providers. Establishing a psychologically safe environment fosters a culture of open communication and collaboration, enabling patients to communicate their ideas, questions, and concerns more effectively, leading to improved communication and collaboration between healthcare providers and patients, ultimately enhancing the patient experience. Additionally, transparent communication about performance by healthcare providers establishes accountability and trustworthiness, which further encourages patient engagement.

Learning from errors

Transparent cultures encourage a “no-blame” approach to mistakes and adverse events [19,20]. When errors occur, organizations that prioritize transparency focus on learning from these incidents rather than assigning blame. This fosters a culture of continuous improvement and reduces the likelihood of similar errors in the future.

Fostering a “no-blame” approach and promoting a culture of learning from errors are key components of transparency in organizations, especially in sectors like healthcare. Transparent organizations view errors as opportunities for improvement rather than assigning responsibility. By encouraging a culture of learning, these organizations can identify the root causes of mistakes and implement changes to prevent similar errors in the future [21]. This continuous improvement mindset helps enhance overall organizational performance.

A recriminate-free culture reduces fear and anxiety among staff. When employees feel safe reporting errors without fear of punishment, they are more likely to be open about their experiences and contribute to the collective learning process. This, in turn, positively impacts staff morale and well-being. Learning from errors is directly linked to improving patient safety. A no-blame approach to errors within a transparent culture is a strategic investment in continuous improvement, staff well-being, patient safety, and overall organizational effectiveness. It is a proactive and positive approach to managing mistakes that can have lasting benefits for both the organization and its stakeholders [22].

Benchmarking and best practices

Transparency enables healthcare organizations to benchmark performance against industry standards and best practices. Benchmarking in healthcare involves comparing performance metrics, processes, and outcomes to established standards or best practices, both internally (within an organization) and externally (against other organizations). Linking benchmarking with transparency can enhance the effectiveness and credibility of healthcare services in several ways. By comparing their outcomes and processes with those of other institutions, organizations can identify opportunities for improvement and adopt successful strategies from their peers [23]. Indeed, transparency in healthcare organizations facilitates benchmarking and the adoption of best practices, contributing to continuous improvement [24].

Transparent organizations can compare their performance metrics, outcomes, and processes with industry benchmarks. This comparison allows healthcare institutions to identify areas where they excel and areas that may require improvement, providing valuable insights for strategic planning. Through transparent reporting and sharing of data, healthcare organizations can pinpoint specific areas where they may lag behind industry benchmarks. This identification of gaps or opportunities for improvement is crucial for organizations committed to enhancing their overall performance [23].

Transparency encourages the sharing of best practices among healthcare organizations. When successful strategies and outcomes are openly communicated, other institutions can learn from these experiences and adopt similar practices, accelerating the diffusion of innovation throughout the industry [25]. Benchmarking against industry standards enables organizations to assess their efficiency and cost-effectiveness [26].

Data-driven decision-making

Transparent organizations use data to inform decision-making processes. Access to real-time data allows healthcare leaders to make informed decisions about resource allocation, process improvements, and interventions that positively impact patient safety and quality of care. Absolutely, data-driven decision-making is a fundamental aspect of transparency in healthcare organizations. Transparent organizations have access to real-time data on various aspects of healthcare operations, including patient outcomes, safety incidents, and quality measures [20]. This allows healthcare leaders to monitor performance continuously and make timely decisions based on current information.

Transparent data aids healthcare leaders in allocating resources effectively. By understanding the current demands and performance of different departments or units, leaders can allocate staff, equipment, and finances where they are most needed, optimizing overall efficiency. Transparency and real-time data empower healthcare leaders to make informed decisions across various aspects of their organizations. This approach contributes to operational efficiency, patient safety, quality improvement, and overall strategic success.

Regulatory compliance and accreditation

Many healthcare regulatory bodies and accrediting agencies require organizations to demonstrate transparency in reporting key performance indicators (KPIs) and outcomes [26]. Compliance with these standards is crucial for maintaining certifications and licenses, driving organizations to prioritize transparency in their operations. Regulatory compliance and accreditation play a significant role in driving healthcare organizations to prioritize transparency. Regulatory bodies and accrediting agencies often mandate the reporting of specific KPIs related to patient safety, quality of care, and organizational performance [27]. Transparent reporting of these KPIs allows organizations to demonstrate their adherence to regulatory standards.

Many regulatory bodies require healthcare organizations to report safety incidents, adverse events, and near misses. Transparent reporting of such incidents not only fulfills regulatory requirements but also contributes to a culture of safety and continuous improvement within the organization. Accreditation bodies often set specific quality measures and outcome benchmarks that healthcare organizations must meet. Transparency in reporting these measures allows organizations to showcase their commitment to quality improvement and compliance with industry standards. Transparency is closely tied to regulatory compliance and accreditation in healthcare. Organizations that prioritize transparency not only meet the requirements set by regulatory bodies and accrediting agencies but also foster a culture of openness and accountability that contributes to the overall improvement of healthcare services.

Patient safety reporting systems

Implementation of systems that allow patients and healthcare providers to report safety concerns or incidents anonymously encourages a culture of transparency [5,10,11]. These reporting systems help identify potential risks and areas for improvement that may not be apparent through routine monitoring. Patient safety reporting systems are crucial tools for promoting transparency and fostering a culture of continuous improvement within healthcare organizations. Patient safety management involves iterative loops for both routine care and critical situations, driving continuous improvement and adaptation in protocols and responses [20], and outlining present safety problems and efforts. Incident reporting systems are crucial tools in various industries, particularly healthcare, aviation, and manufacturing, to ensure safety, quality control, and continuous improvement. These systems allow employees to report accidents, near misses, and unsafe conditions, providing valuable data for identifying trends, preventing future incidents, and improving overall safety protocols. These facilitate the early identification of potential risks before they escalate into serious issues, enabling proactive intervention.

Anonymity in reporting encourages individuals to share information without fear of reprisal. Healthcare providers may be more willing to report errors, near misses, or unsafe conditions, contributing to a more comprehensive understanding of potential risks. The implementation of reporting systems sends a clear message that the organization values transparency and openness [5]. This contributes to the development of a safety culture where individuals feel empowered to communicate openly about safety concerns and contribute to the overall improvement of patient care. Patient safety reporting systems capture not only adverse events but also near misses-incidents that had the potential to cause harm but were prevented. Learning from near misses is a valuable aspect of a reporting system, as it provides insights into vulnerabilities that can be addressed to prevent future incidents. The data collected through reporting systems can be analyzed to identify patterns, root causes, and areas for improvement [28]. Timely reporting of safety concerns allows organizations to take prompt corrective action, mitigating potential risks. This proactive approach helps prevent the recurrence of similar incidents and contributes to overall patient safety. These systems contribute to early risk identification, continuous improvement, and the development of a culture that prioritizes patient safety and well-being.

The paradigm shift in healthcare toward patient safety and quality improvement necessitates a comprehensive understanding and proactive management of adverse events. Traditionally, the focus has been on departments perceived as “dangerous,” but there is a growing recognition that incidents reported from various departments, particularly those related to accident extraction power and transparency, are invaluable in identifying potential risks and improving safety culture [29,30]. The incident reporting system serves as a vital tool in this endeavor, offering insights into representative patient safety issues and enabling the identification of both near miss incidents and adverse events. The accumulation of near misses, even those seemingly minor in impact, highlights potential risks that could escalate into adverse events if not addressed proactively. Moreover, there is a demonstrated positive correlation between the number of incident reports and an enhanced safety culture within healthcare organizations.

Medical doctors play a pivotal role in reporting adverse events and facilitating coordinated treatment for severe injuries and chronic conditions, while also contributing to organizational transparency [31]. The differential reporting patterns among specialties underscore the need for tailored strategies for addressing patient safety concerns. While doctors often report harm incidents, nurses frequently report attempted and harmless cases, emphasizing the importance of a multifaceted approach to incident reporting [5,31]. As safety managers, the goal is to address adverse events promptly while leveraging serious incidents to drive systemic improvements across the hospital. The increase in reporting from medical doctors signals a shift toward greater organizational transparency and underscores the dynamic efforts required for continuous quality improvement in healthcare [5,32]. Overall, the evolving landscape of incident reporting reflects a commitment to seamless improvement in patient safety and care, with stakeholders across healthcare actively engaged in driving positive change and fostering a culture of safety and transparency. This ongoing effort signifies a significant step forward in ensuring the highest standards of quality and safety in healthcare delivery.

## Conclusions

Transparency in healthcare organizations is foundational for establishing a culture of patient-centered care where patients are valued, informed, and actively involved in their health decisions. By promoting open communication about performance, outcomes, and processes, healthcare organizations enhance accountability, trust, and patient engagement. Patient-centered care is prioritized through transparency, fostering shared decision-making and understanding between providers and patients. Moreover, psychological safety is crucial in supporting transparency, enabling healthcare professionals to report safety concerns and incidents without fear of retribution.

Effective patient safety reporting systems are instrumental in this regard, allowing anonymous reporting and facilitating early risk identification, continuous improvement, and regulatory compliance. These systems encourage a culture of openness, learning from near misses, and addressing systemic issues and human errors, thereby aligning with ethical principles and potentially reducing legal challenges. Overall, organizational transparency in healthcare drives accountability, patient engagement, and continuous improvement, ultimately contributing to safer care and the ongoing enhancement of patient safety and quality.
